# Quadriceps, hamstring and patella tendon autografts for primary anterior cruciate ligament reconstruction demonstrate similar clinical outcomes, including graft failure, joint laxity and complications: A systematic review with meta‐analysis of randomised controlled trials

**DOI:** 10.1002/ksa.12755

**Published:** 2025-07-18

**Authors:** Ty White, Matthew Castro, Lucas Antonio, Wayne Hing, Francois Tudor, Larissa Sattler

**Affiliations:** ^1^ Institute of Health and Sport Bond University Gold Coast Queensland Australia; ^2^ Gold Coast Hospital and Health Service Gold Coast Queensland Australia

**Keywords:** anterior cruciate ligament reconstruction, bone‐patellar tendon‐bone, hamstring tendon, quadriceps tendon

## Abstract

**Purpose:**

Graft failure following anterior cruciate ligament reconstruction (ACLR) remains a significant challenge, driving discussion for optimal graft choice. Traditionally, hamstring tendon (HT) and bone‐patella tendon‐bone (BPTB) autografts have been favoured for ACLR. Recently, quadriceps tendon (QT) usage has increased. This systematic review with meta‐analysis aims to investigate the available evidence for QT compared to HT and BPTB autografts in terms of graft failure, clinical outcomes, functional outcomes and patient‐reported outcome measures (PROMs).

**Methods:**

Five databases (PubMed, Cochrane, Embase, SPORTDiscus and CINAHL) were searched to identify randomised controlled trials comparing QT versus HT or BPTB autografts. Outcomes included graft failure, donor site morbidity, complications, reoperations, clinical measures, functional outcomes, and PROMs. Risk of bias was assessed using the Cochrane risk‐of‐bias tool (RoB2). Meta‐analysis using a random effects model was conducted to determine the pooled risk ratio (RR) or mean difference (MD) with 95% confidence intervals (CIs), and certainty of evidence was evaluated via the GRADE approach.

**Results:**

Twelve RCTs consisting of 636 patients were included. Meta‐analysis revealed no differences in graft failure rates (RR = 1.00; 95% CI = 0.97–1.04; *p* = 0.83; *I* ^2^ = 0%; high certainty). QT autografts demonstrated lower donor site morbidity when compared to HT and BPTB autografts (RR = 1.45; 95% CI = 1.24–1.70; *p* < 0.001; *I* ^2^ = 0%; high certainty). No differences were observed in joint laxity or PROM scores at 12 and 24 months, except for Knee injury and Osteoarthritis Outcome Score favouring HT and BPTB at 12 months (RR = 2.49; 95% CI = −4.69 to −0.28; *p* = 0.03; *I* ^2^ = 0%; very low certainty).

**Conclusion:**

QT autografts have similar outcomes for graft failure, laxity and PROMs compared to HT and BPTB; however, QT autografts may have lower donor site morbidity. While each graft type is associated with specific complications and post‐operative strength deficits, all three remain viable options for ACLR.

**Level of Evidence:**

Level I, systematic review of Level I randomised controlled studies.

**Abbreviations**
ACL: anterior cruciate ligament
ACLR: anterior cruciate ligament reconstruction
AP: anteroposterior
BMI: body mass index
BPTB: bone‐patella tendon‐bone
CI: confidence interval
CSA: cross‐sectional area
GRADE: Grading of Recommendations Assessment, Development and Evaluation
HQ: hamstrings:quadriceps
HT: hamstring tendon
IKDC: International Knee Documentation Committee
KOOS: Knee injury and Osteoarthritis Outcome Score
KT‐1000: Knee Laxity Testing Device
LSIs: Limb symmetry indices
MD: mean difference
PICOS: Population, intervention, comparison, outcome and study design
PRISMA: Preferred Reporting Items for Systematic Reviews and Meta‐Analyses
PROMs: patient‐reported outcome measures
QT: quadriceps tendon
RCT: randomised controlled trial
RoB2: Cochrane risk‐of‐bias tool
RR: risk ratio
SD: standard deviation

## BACKGROUND

Anterior cruciate ligament (ACL) injuries remain one of the most common knee injuries among athletes [8, 12], with approximately 400,000 ACL reconstruction (ACLR) surgeries performed annually in the United States, and these numbers continue to rise each year [18, 68, 104]. While largely regarded as an effective procedure, reported failure rates range from 2% to 10%, which incurs significant morbidity, loss of function and substantial direct and indirect costs [18, 21, 55, 59]. Different rates of graft failure are also observed depending on the specific graft chosen for the primary reconstruction [44, 51, 55, 79, 84].

With the significant increase in ACL injuries and subsequent surgical intervention, there is ongoing debate regarding the optimal graft choice for ACLR. Traditionally, hamstring tendon (HT) and bone‐patellar tendon‐bone (BPTB) autografts have been preferred [17, 99]. Current worldwide trends indicate a predominant use of HT autografts, accounting for 80% of isolated ACLR, with 16% of ACLR utilising BPTB autografts and just 2.5% consisting of quadriceps tendon (QT) autografts [99]. However, this trend appears to be shifting, as QT grafts have gained popularity in recent years as a viable alternative [5, 81, 93]. While HT autografts offer the advantage of maintaining the knee extensor mechanism and reportedly reduced post‐operative knee pain compared to BPTB autografts [12, 17, 26], increasing evidence suggests HT autografts have a greater potential for failure when compared to both BPTB and QT alternatives [3, 31, 43, 72].

With respect to BPTB autografts, earlier graft incorporation is observed, resulting in potentially faster rates of return to sport [20, 62] and probable lower failure rates compared to HT grafts [3, 17]. Despite these advantages, BPTB autograft harvesting has a known risk of subsequent anterior knee pain reportedly as high as 17%, alongside a risk of patella fracture and possibly patellofemoral osteoarthritis [46, 103]. The increased utilisation of QT graft is due to the inference that the failure rate will be lower due to the robust QT autograft structure, with a greater cross‐sectional area (CSA) than an HT graft and the possibility to utilise a bone block if desired [3, 43]. However, despite the reported reduced incidence of anterior knee pain compared to BPTB [1, 17, 69], there remains some concern about the likelihood of prolonged knee extensor weakness [17, 69, 89, 90].

It is recognised that autograft selection can significantly influence failure rate and both functional and clinical outcomes for patients undergoing ACLR. To achieve optimal post‐operative outcomes, an individualised approach should be taken, considering each patient's anatomy, activities and goals [10, 54], with the chosen autograft best suited to the patient's sports and employment to allow the efficient return to full function [39, 54]. Despite several previous systematic reviews examining ACLR graft choice [17, 50, 69, 80, 105], there remains a lack of consensus regarding optimal autograft selection. This systematic review with meta‐analysis addresses this knowledge gap through its specific focus on comparing the less utilised QT autograft against traditional options, the exclusion of non‐randomised evidence, and its comprehensive analysis across multiple outcome domains. By systematically evaluating graft failure, complications, clinical outcomes, objective functional measures and patient‐reported outcomes (PROMs), this review aims to build on the existing evidence base for surgeons seeking to optimise autograft selection for individual patients undergoing ACLR.

## METHODS

### Protocol design

This systematic review with meta‐analysis was performed and reported according to the Preferred Reporting Items for Systematic Reviews and Meta‐Analyses (PRISMA) guidelines [74]. The study protocol was prospectively registered with Open Science Framework (https://doi.org/10.17605/OSF.IO/JN2G6) on 7 August 2024 [101].

### Eligibility criteria

Studies meeting the PICOS (population, intervention, comparison, outcome and study design) were included in this review:

1. Population: Individuals of any age or sex who have undergone primary ACLR surgery with or without concomitant meniscal repair.
2. Intervention: Primary ACLR surgery using QT autograft (with or without bone block).
3. Comparison: HT autograft and/or BPTB autograft.
4. Outcomes of interest: Graft failure, defined as tear of the graft (primary outcome), donor site morbidity, complications, reoperations, clinical measures, objective functional outcomes and PROMs.
5. Study design: Randomised controlled trials (RCTs).

#### Exclusion criteria

Not of RCT study design, multi‐ligament reconstructive surgery, revision ACLR, utilisation of allograft, studies outside of the specified search timeline, unavailable in full text (example conference abstract only), and study protocols. No language restrictions were applied; however, due to surgical advancements in graft harvest and fixation techniques, a 20‐year limit was applied to the search.

### Data sources and search strategy

A comprehensive search from 26 July 2004 to 26 July 2024 was completed within each of the following five databases: PubMed, Cochrane, Embase, SPORTDiscus and CINAHL. An initial search strategy was developed by authors (TW, MC, LS and LA) with the assistance of a university Health Sciences and Medicine Faculty librarian using the following key terms: ‘Anterior Cruciate Ligament’, ‘Hamstring’, ‘Semitendinosus’, ‘Gracilis’, ‘Patella Tendon’, ‘Bone Patella Tendon Bone’ and ‘Quadriceps’ as well as synonyms and Boolean operator terms. Utilisation of Medical Subject Headings terms and Title and Abstract fields were applied to the search. The search strategy was piloted on PubMed to ensure that key studies were identified within the search. Once verified, the search strategy was modified to suit each database using Polyglot Search Translator on the Systematic Review Accelerator [14]. A complete list of search strategies for each database is reported in Appendix S1.

### Study selection

All search results from databases were imported to EndNote. Duplicates were removed using the De‐Duplicator tool on the Systematic Review Accelerator [25]. Initial title and abstract screening were performed independently by two authors (MC and LA) via Screenatron on Systematic Review Accelerator [13]. Following title/abstract screening, the same two authors performed full text screening independently. Full‐text studies that did not meet the eligibility criteria were excluded, with reasons reported. Discrepancies on the eligibility of full‐text studies were resolved by a third author (TW) to ensure consensus was met.

### Data extraction

Two authors (MC and LA) independently extracted data using a standardised data extraction table on Excel adapted from the Cochrane Collaboration Data Collection Template (Cochrane Handbook for Systematic Reviews of Interventions) [53]. Baseline characteristics were extracted from the included studies: author(s), year of publication, country, participant characteristics (sex, age), graft type, any reported complications and reoperations, along with complete study inclusion and exclusion criteria. For continuous outcomes, means and standard deviations were extracted, while for categorical data, counts and percentages were collected. Key outcomes of interest, including graft failure, surgical complications, clinical outcomes, functional outcomes, PROMs and assessment timepoints, were also extracted.

### Assessment of risk of bias

A study‐level assessment of the risk of bias in the selected studies was carried out independently by two authors (MC and LA) using the Cochrane risk‐of‐bias tool (RoB2) for RCTs [91]. The tool is structured into five domains of bias, each examining different aspects of trial design, conduct and reporting [91]. Within these domains, signalling questions are used to gather information about specific elements of the trial that are relevant to assessing bias.

An algorithm generates a proposed judgement (low, some concerns or high) based on the responses to these signalling questions [91]. Given the potential for discrepancy between the two authors (MC and LA), a third author (TW) reviewed to achieve a consensus if there were disagreements. Following the scoring of the risk of bias appraisal of each study, the Kappa coefficient of inter‐rater reliability was calculated. Values ranging from 0.81 to 1.00 were interpreted as almost perfect, 0.61–0.80 as substantial, 0.41–0.60 as moderate, 0.21–0.40 as fair and 0.0–0.2 as slight [91].

### Synthesis and statistical analysis

A quantitative meta‐analysis was completed using Review Manager software (RevMan 5.4) to compare the overall effect between autograft options on outcomes from eligible studies. Outcomes were eligible for meta‐analysis if they were present in two or more studies and assessed at similar time points. Dichotomous variables were extracted as the absolute number and pooled by utilising the Mantel‐Haenszel method and presented as risk ratio (RR) with a 95% confidence interval (CI). Continuous variables were extracted as the mean and standard deviation (SD), pooled by using the inverse variance weighting method and presented as the mean difference (MD) with the 95% CI. Risk difference (RD) was calculated with absolute difference in event rates between two groups with 95% CI. Random‐effect models were used for all analyses due to the clinical and methodological heterogeneity of studies. Statistical heterogeneity of study outcomes was determined via visual inspection of forest plots and by the *I* ^2^ index, values from 50% to 100% are observed as having considerable heterogeneity [38]. Results were considered statistically significant, where *p* < 0.05. In cases where it was not possible to undertake meta‐analysis, such as when only one study reported on an outcome, the data were extracted into tabular or narrative synthesis and individual results were presented.

### Determining certainty of evidence

The Grading of Recommendations Assessment, Development and Evaluation (GRADE) approach was utilised to evaluate the certainty of evidence and overall confidence in the cumulative estimate [32]. In this approach, RCTs are initially considered to provide high‐certainty evidence. However, the certainty of pooled estimate may be downgraded based on specific factors, including risk of bias (methodological limitations), inconsistency (unexplained heterogeneity between studies), indirectness (whether evidence addresses the specific research question), imprecision (CI width and sample size adequacy), and publication bias (whether selective reporting has distorted the available evidence pool). Each domain receives a judgement of ‘not serious’ (no downgrade), ‘serious’ (−1 level) or ‘very serious’ (−2 levels), with cumulative downgrades across domains determining the final certainty rating from high to very low (e.g., high certainty evidence with two ‘serious’ concerns becomes low certainty). Outcome was categorised into one of four levels of certainty: ‘very low’, ‘low’, ‘moderate’ or ‘high’.

## RESULTS

### Study selection

**Figure 1:**
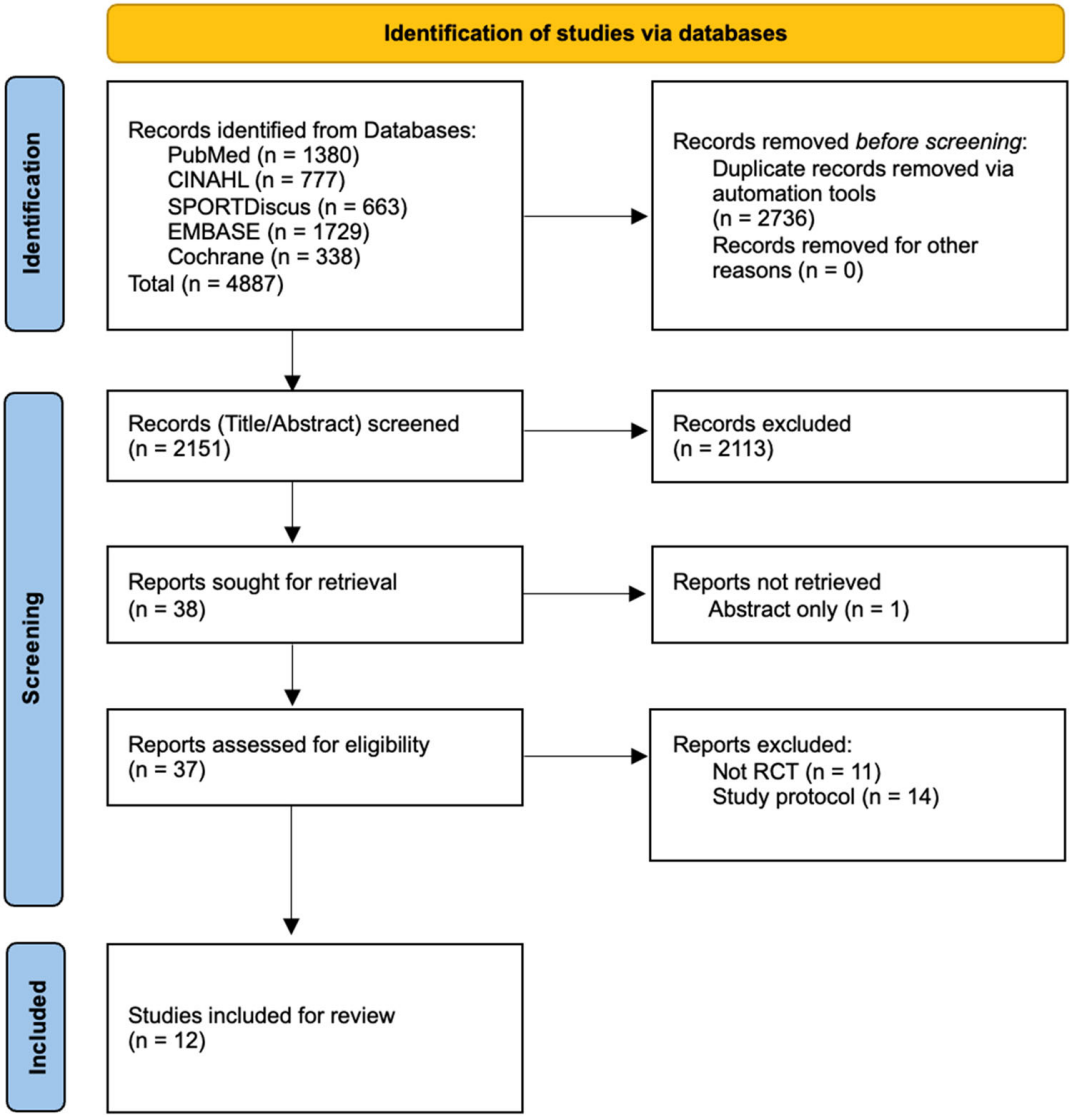
PRISMA flow diagram of the screening process, including selection and removal of studies. PRISMA, Preferred Reporting Items for Systematic Reviews and Meta‐Analyses; RCT, randomised controlled trial.

The initial search identified 4887 records, after the removal of duplicates via Systematic Review Accelerator Deduplicator [25], 2151 remained. Following title and abstract screening, 38 reports were eligible for screening at full text. After applying eligibility criteria, 12 studies [6, 11, 23, 40, 56, 60, 63, 64, 88, 94, 97, 100] were included in this systematic review (Figure 1).

### Assessment of risk of bias

**Figure 2:**
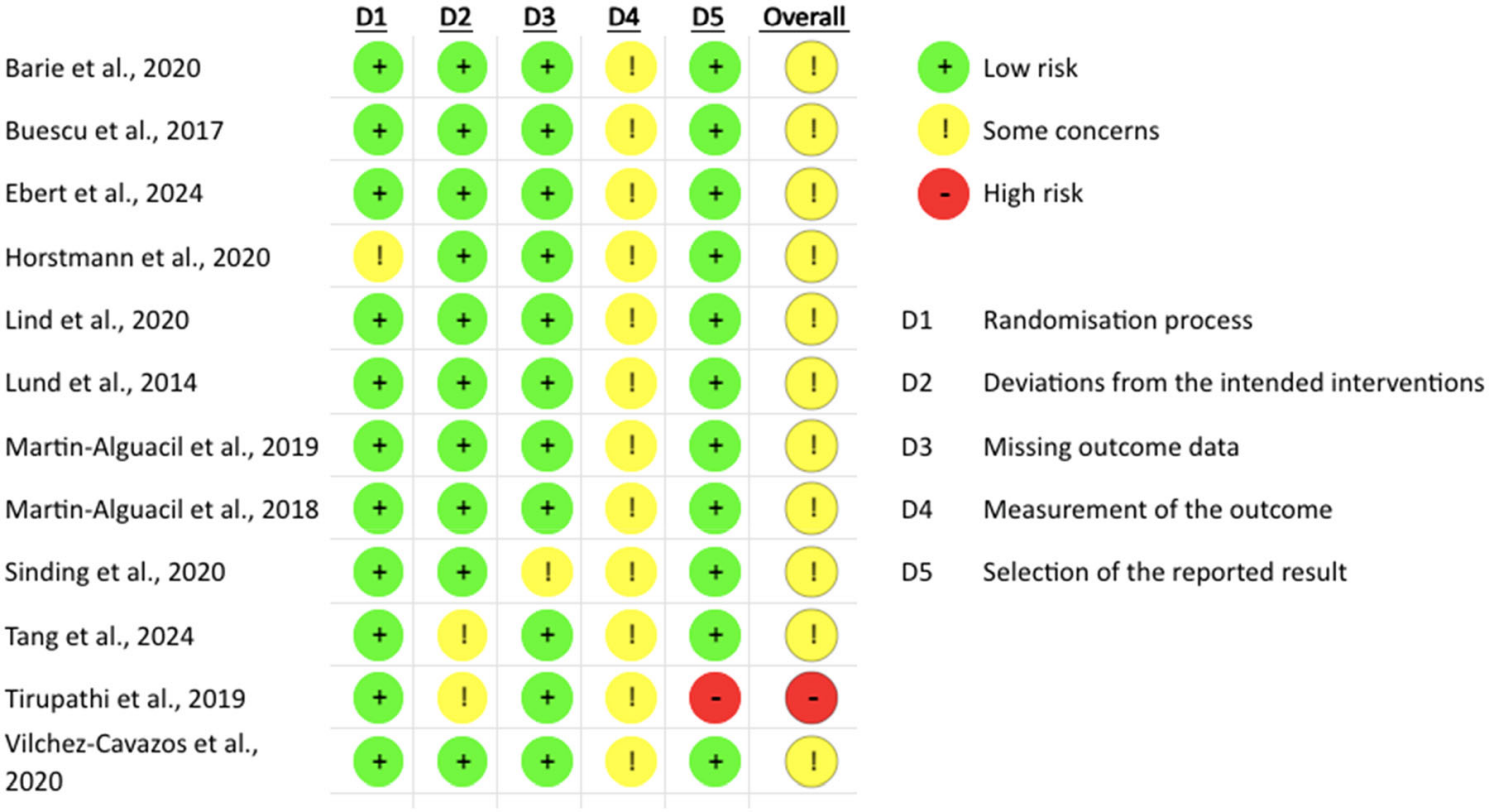
Risk‐of‐bias tool 2 (RoB2) assessment.

Upon assessment of risk of bias (Figure 2), eight studies showed some concerns [6, 11, 23, 56, 60, 63, 64, 100] regarding outcome measurement due to the inability to blind surgeons. Three studies [40, 88, 94] showed some concerns regarding missing data, intervention deviations, and randomisation processes. One study [97] was judged high risk, primarily due to the selection of reported results, as it failed to report all outcomes from the study protocol. All twelve studies [6, 23, 40, 56, 60, 63, 64, 88, 94, 97, 100] showed some concerns within domain four of the RoB2 due to difficulties blinding patients and assessors. The authors agreed that due to blinding difficulties for the selected surgical interventions, this domain would not factor into the overall risk judgement [7]. Four articles [40, 88, 94, 97] found differences in outcome measures, while eight low‐risk articles [6, 11, 23, 56, 60, 63, 64, 100] reported similar outcomes, improving evidence quality. Interrater reliability between authors (MC and LA) was strong, with a Kappa coefficient of 0.87, regarded as almost perfect agreement [52].

### Certainty of meta‐analysis findings

The certainty of evidence was rated for all outcomes within this review by utilising the GRADE approach (Table 1). The highest level of certainty for any outcome was considered ‘high’ for graft failure, donor site morbidity, pivot shift and Tegner scores. The KT‐1000 was considered ‘moderate’. All other outcomes assessed were considered ‘low’ due to moderate heterogeneity, large CI and moderate sample sizes with no effect. Publication bias was undetected via forest plot inspection; however, the reliability of this was limited, as meta‐analyses included fewer than 10 studies.

**Table 1:** Findings and GRADE evidence profiles of randomised controlled trials.

Outcome (no. of studies)	No. of patients	Risk of bias	Inconsistency	Indirectness	Imprecision	Publication bias	Certainty
Graft failure (6)	429	Not serious	Not serious	Not serious	Not serious	Undetected	⊗⊗⊗⊗ High
Donor site morbidity (3)	210	Not serious	Not serious	Serious^a^	Not serious	Undetected	⊗⊗⊗໐ Moderate
KT‐1000 (6)	424	Not serious	Not serious	Not serious	Serious^b^	Undetected	⊗⊗⊗໐ Moderate
Pivot shift (3)	195	Not serious	Serious^c^	Serious^a^	Serious^d^	Undetected	⊗⊗໐໐ Low
IKDC (5)	353	Serious^e^	Serious^c^	Not serious	Very serious^f^	Undetected	⊗໐໐໐ Very Low
Lysholm (5)	291	Serious^e^	Not serious	Not serious	Very serious^f^	Undetected	⊗⊗໐໐ Low
KOOS (4)	302	Serious^e^	Serious^c^	Not serious	Very serious^f^	Undetected	⊗໐໐໐ Very Low
Tegner (3)	271	Not serious	Not serious	Not serious	Serious^f^	Undetected	⊗⊗⊗⊗ High

Abbreviations: CI, confidence interval; GRADE, Grading of Recommendations Assessment, Development and Evaluation; IKDC, International Knee Documentation Committee; KOOS, Knee injury and Osteoarthritis Outcome Score; RoB2, Cochrane risk‐of‐bias tool.

^a^ Differences in applicability of interventions.

^b^ Wide CI showing no effect.

^c^ Moderate or substantial heterogeneity across studies.

^d^ Wide CI showing no effect. Sample size is small.

^e^ ROB2 assessment = variations in blinding within included studies.

^f^ CI is very wide showing no effect. Sample size is moderate.

### Participant demographics

The 12 included studies evaluated 636 patients: 319 received QT autografts, 262 received HT autografts and 55 received BPTB autografts. Two studies [63, 88] were longer‐term follow‐ups of previous study groups, thus only the initial studies' participant demographics were included to avoid duplication. Age and sex distribution data were available from eight studies. The weighted mean ages were 26.9 years for the QT group, 27.3 years for the HT group and 30.8 years for the BPTB group [6, 11, 23, 40, 56, 60, 63, 64, 88, 94]. Sex distribution analysis with weighted averages [6, 11, 23, 40, 56, 60, 63, 64, 88, 100] showed that the QT group included 169 males (67.3%) and 82 females (32.6%), the HT group had 112 males (58%) and 82 females (42%), and the BPTB group comprised 38 males (69%) and 17 females (31%). Across all groups, the total distribution was 319 males (64%) and 181 females (36%). Follow‐up periods of studies ranged from the day of surgery to 10 years post‐operatively. Detailed demographic characteristics for individual studies, along with all assessment time points, are available in Appendix S2. Study inclusion and exclusion criteria are detailed in Appendix S3.

### Primary outcome

#### Graft failure

**Figure 3:**
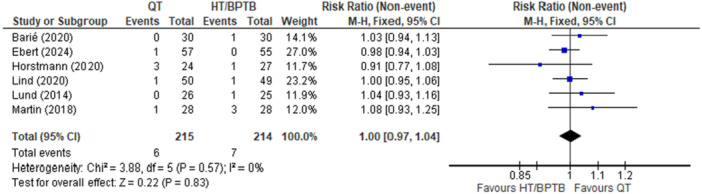
Meta‐analysis comparing graft failure in hamstring tendon (HT) and bone‐patellar tendon‐bone (BPTB) versus quadriceps tendon (QT). CI, confidence interval.

A comparison of QT versus HT and/or BPTB autografts in regard to graft failure was reported in six studies [6, 23, 40, 56, 60, 64], two studies compared QT to BPTB [6, 60], while four studies compared QT to HT [23, 40, 56, 64]. Results can be found in Appendix S4. The overall pooled effect demonstrated that QT autograft showed no significant difference when compared to HT and BPTB autografts (RR = 1.00; 95% CI = 0.97–1.04; *p* = 0.83; *I* ^2^ = 0%; high certainty; Figure 3). Graft failure occurred in 2.79% of patients who received QT autografts compared to 3.14% and 3.6% of patients who received HT and BPTB grafts, respectively. Graft failures were documented across different time points for each autograft type. For QT autografts, two failures occurred within 12 months [56, 64], one between 12 and 24 months [23] and three at unspecified time points [40]. HT autografts experienced four failures within 12 months [60, 69] and one at an unspecified time point [42]. BPTB autografts had two failures: one within 12 months [6, 56, 64] and another between 12 and 24 months [60].

### Secondary outcomes

#### Complications and reoperations

Six studies reported post‐operative complications excluding graft failure following ACLR [6, 23, 40, 56, 60, 94]. Arthrofibrosis complications were reported in three studies [6, 23, 56], described in seven QT, three BPTB and seven HT autograft ACLR. Two studies [56, 60] reported local implant irritation via protruding tibial screws once in each autograft type. Post‐operative infections occurred twice with QT autografts [40, 60], while the infrapatellar branch of the saphenous nerve palsy affected four HT autografts [94]. Reoperations included scar tissue debridement (QT and BPTB groups) [23] in both the QT and BPTB groups, meniscal repairs (two QT, four HT) [6, 23, 60] osteochondral autologous transplant (one QT) [23], partial meniscectomy (one BPTB) [60], plica resection (one QT) participant [23], tibial screw removal (all groups) [56, 60], staple removal (one HT) [23] and notch debridement (one QT) [23]. Meta‐analysis of complications was not possible due to reporting variability. A complete list of secondary outcomes is listed in Appendix S5, and complications are reported in Appendix S6.

### Donor site morbidity

**Figure 4:**
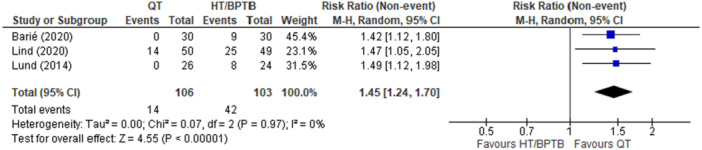
Meta‐analysis comparing donor site morbidity in hamstring tendon (HT) and bone‐patellar tendon‐bone (BPTB) versus quadriceps tendon (QT). CI, confidence interval.

A comparison of QT versus HT and/or BPTB autografts on donor site morbidity was reported in five studies [6, 23, 56, 60, 100]. Three studies [6, 56, 60] were eligible for pooled analysis; these three studies reported on questions 9c and 9d of International Knee Documentation Committee (IKDC) scores [6], donor site morbidity scores [56], and harvest site pain scores [60]. Of the three studies included in the pooled analysis, one study compared QT versus HT [56], and two studies compared QT versus BPTB [6, 60]. The overall pooled effect demonstrated that QT autograft was associated with a significant reduction in donor site morbidity when compared with HT and BPTB autografts (RR = 1.45; 95% CI = 1.24–1.70; *p* < 0.001; *I* ^2^ = 0%; high certainty; Figure 4). Donor site morbidity occurred in 13.2% of patients with QT autografts compared to 40.8% of patients with either HT or BPTB autografts (RD, −27.6%). The two studies that were not included within the pooled analysis [23, 100] were excluded as they presented their donor site morbidity data as continuous variables without specifying event frequencies. Both studies examined QT versus HT autografts and demonstrated no significant differences with respect to donor site morbidity within 12 months post‐operatively.

### Clinical outcomes

**Figure 5:**
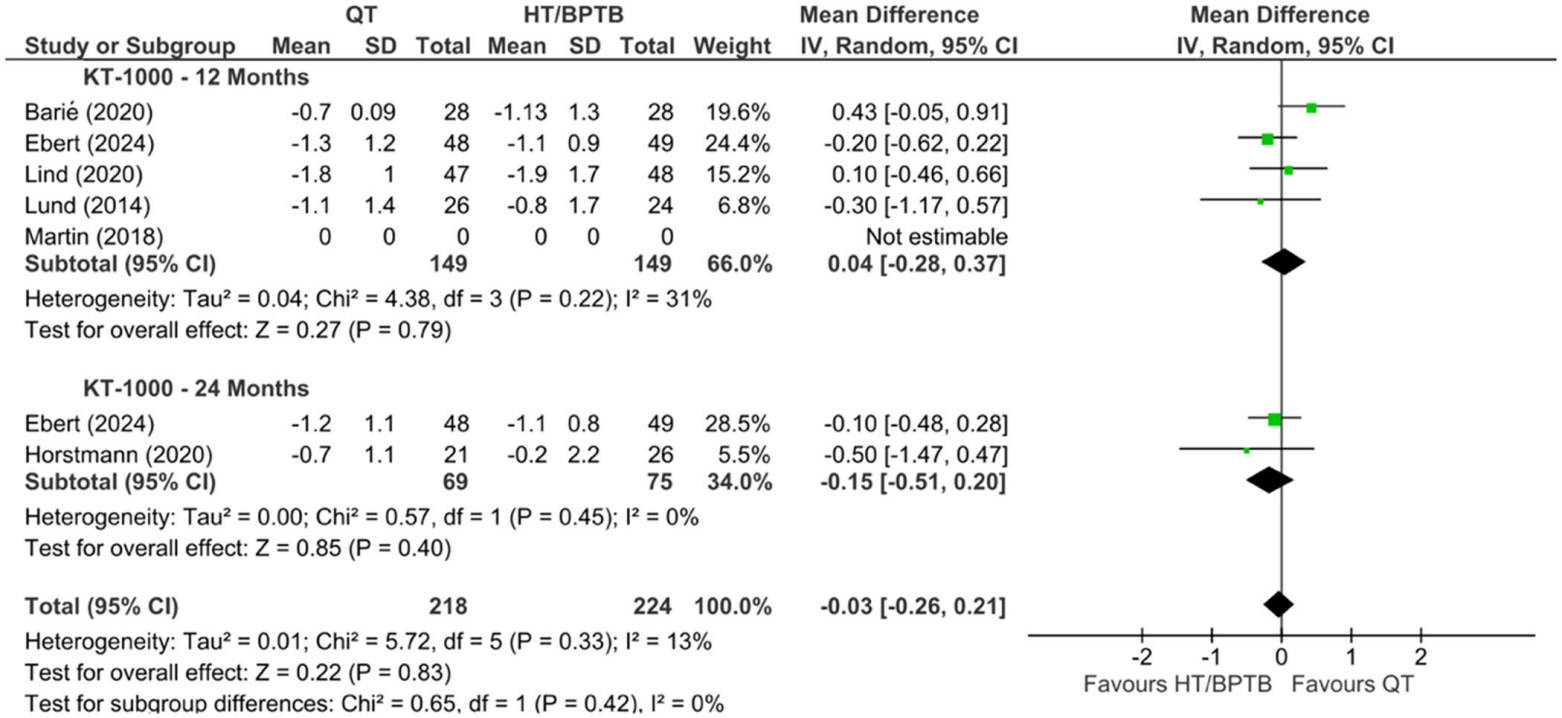
Meta‐analysis comparing KT‐1000 scores in hamstring tendon (HT) and bone‐patellar tendon‐bone (BPTB) versus quadriceps tendon (QT) at 12 and 24 months. CI, confidence interval; KT‐1000, Knee Laxity Testing Device; SD, standard deviation.

A comparison of joint laxity between QT versus HT and/or BPTB autograft with the KT‐1000 Knee Arthrometer was reported in six studies at 12‐ and 24‐month periods. Of the six studies that reported on KT‐1000 as a clinical outcome [6, 23, 40, 56, 60, 64], four compared QT to HT [23, 40, 56, 64] and two compared QT to BPTB [6, 60]. Across the six studies reporting on KT‐1000, no significant differences were observed between QT and HT and BPTB. The pooled effect indicated no significant differences in joint laxity between QT and other graft types (HT and BPTB) at both 12 and 24 months post‐surgery (MD = −0.03; 95% CI = −0.26 to 0.2; *p* = 0.83; *I* ^2^ = 13%; moderate certainty; Figure 5).

**Figure 6:**
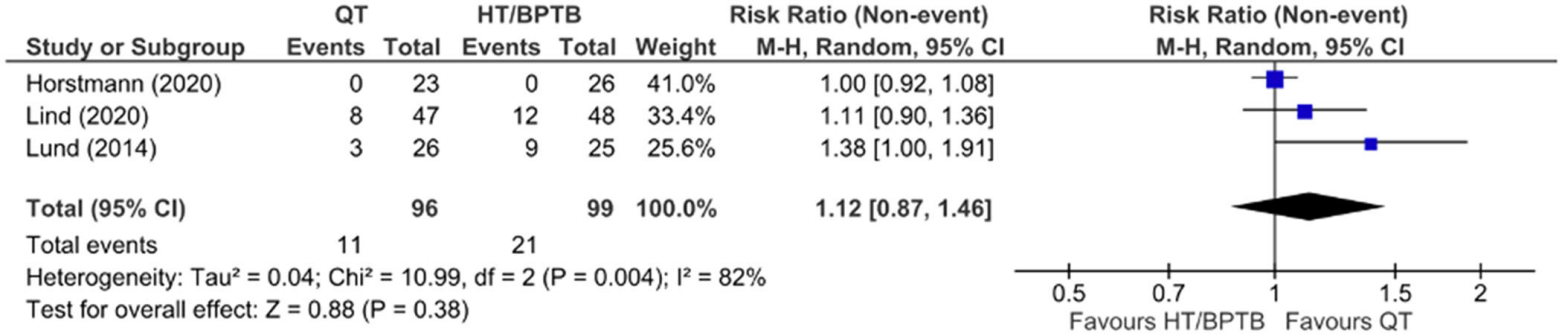
Meta‐analysis comparing positive pivot shift test in hamstring tendon (HT) and bone‐patellar tendon‐bone (BPTB) versus quadriceps tendon (QT) at 12 months. CI, confidence interval.

A comparison of joint laxity between QT versus HT and/or BPTB autografts in relation to positive pivot shift test results was reported in three studies at 12–24 months. Between the three studies [56, 60] reporting on the pivot shift test, the overall pooled effect demonstrated no significant difference between QT versus HT and BPTB (RR = 1.12; 95% CI = 0.87–1.46; *p* = 0.38; *I* ^2^ = 82%; moderate certainty; Figure 6).

### Objective functional outcomes

Four studies [23, 64, 88, 94] reported on objective functional outcomes through the measurement of strength scores. In Ebert et al. [23], patients who received QT autografts had significantly greater hamstring strength limb symmetry indices (LSIs) at 6, 12 and 24 months (*p* < 0.05), while patients with HT autografts had significantly greater quadriceps strength LSIs at 6 and 12 months (*p* < 0.05) [23]. Martin‐Alguacil et al. [64], found that patients who received ACLR with QT autograft showed a significantly greater isokinetic hamstring:quadriceps (HQ) ratio when compared to patients who received ACLR with an HT autograft at 12 months (*p* < 0.01) [64]. Sinding et al. [88] report that HQ ratios were also significantly higher for the QT autograft group when compared to the HT autograft group. Furthermore, at 1 year post‐ACLR, it was found that the QT autograft group had notable impairments involving knee extensor muscle strength, compared to the HT autograft group, which experienced moderate impairments of knee extensor and flexor muscle strength [88]. Tang et al. [94] found that at 24 months, the QT autograft group had a significantly greater HQ ratio when compared to the HT autograft group (*p* < 0.05). A meta‐analysis involving objective strength measures was not performed due to insufficient and inconsistent reporting of strength outcomes.

### Patient‐reported outcomes

**Figure 7:**
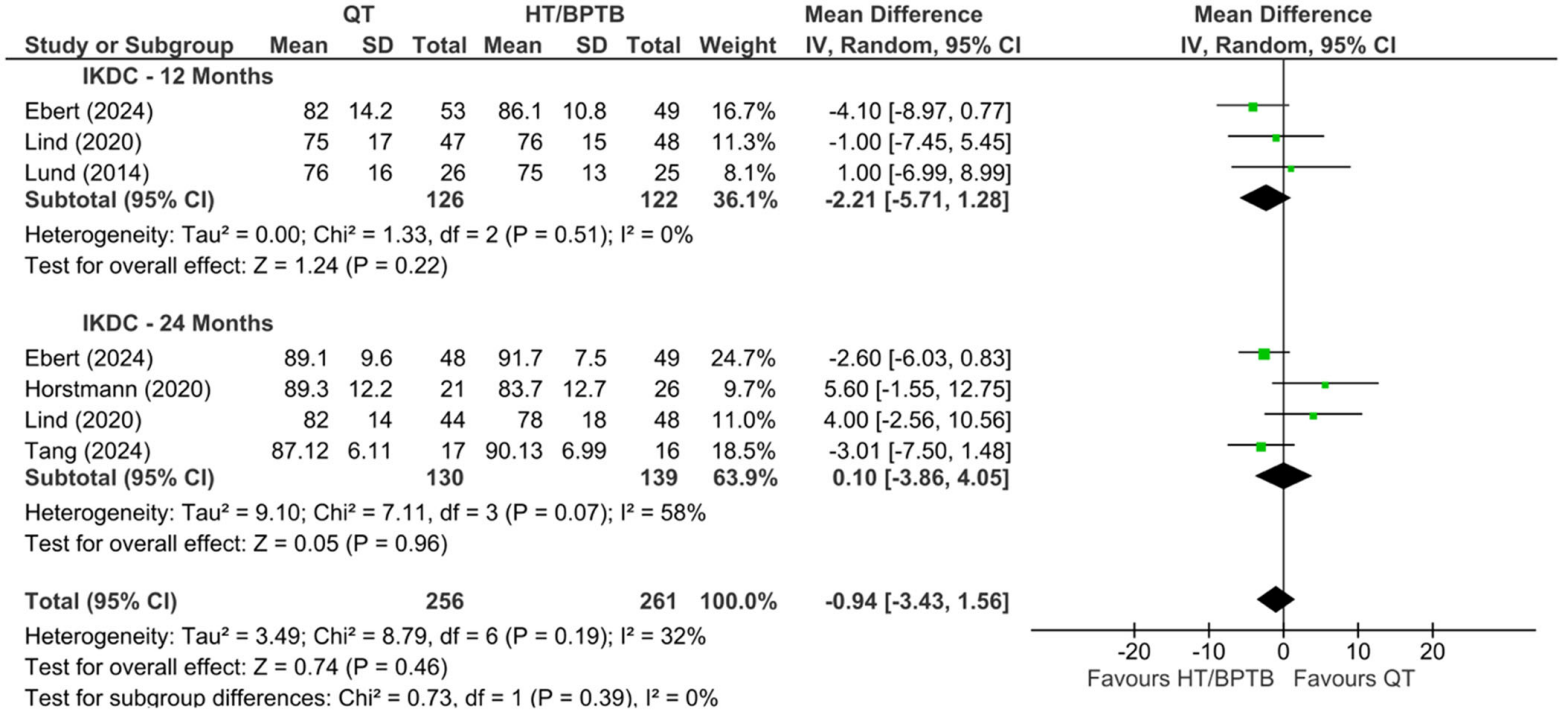
Meta‐analysis comparing IKDC scores in hamstring tendon (HT) and bone‐patellar tendon‐bone (BPTB) versus quadriceps tendon (QT) at 12 and 24 months.

**Figure 8:**
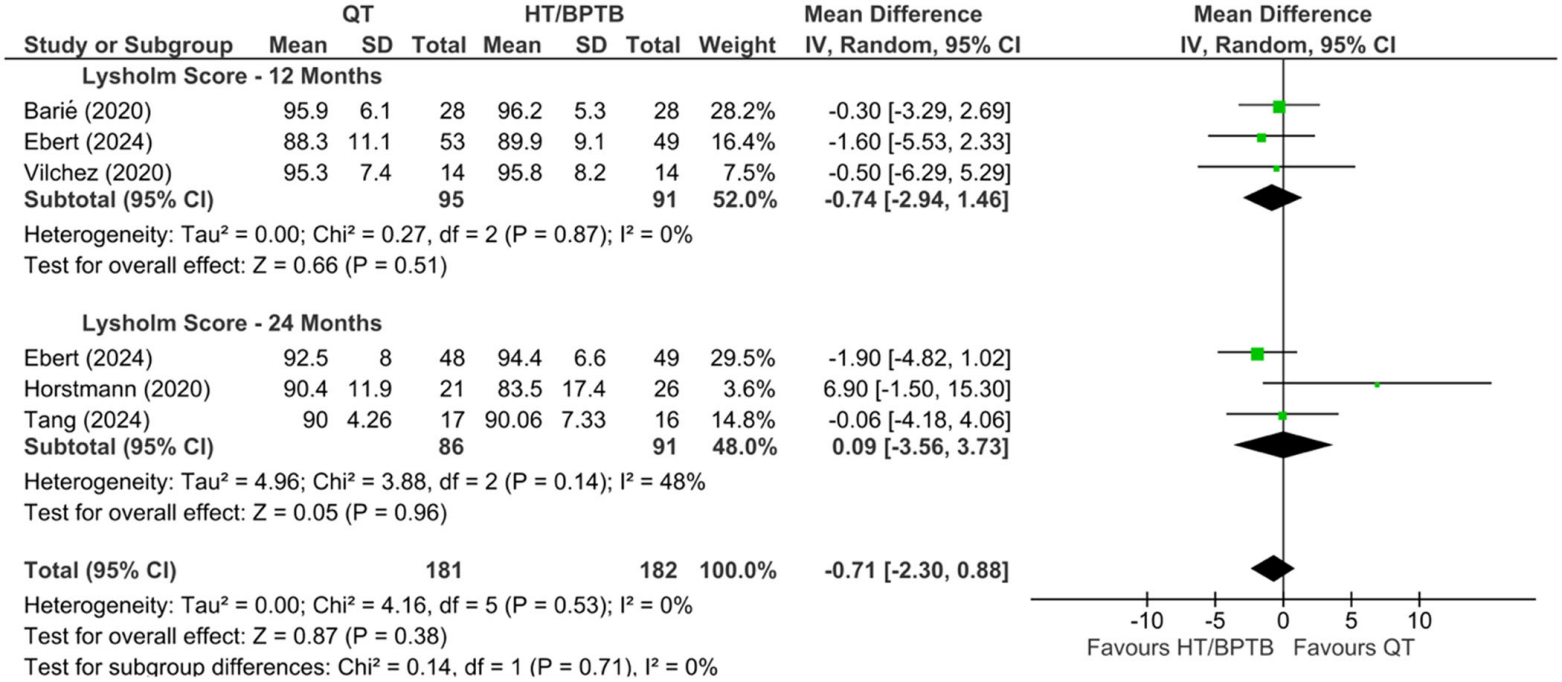
Meta‐analysis comparing Lysholm scores in hamstring tendon (HT) and bone‐patellar tendon‐bone (BPTB) versus quadriceps tendon (QT) at 12 and 24 months.

A comparison of QT versus HT and/or BPTB autografts for IKDC and Lysholm scores was performed in seven studies collectively [6, 23, 40, 56, 60, 94, 100], which reported patient outcomes at both 12 and 24 months. Across all studies reporting on IKDC and Lysholm scores, no significant differences were observed between QT and HT or BPTB. The overall effect showed that QT was associated with similar outcomes on the IKDC and Lysholm scored when compared to HT and BPTB with no significant difference at both the 12 and 24 months (MD = −0.94; 95% CI = −3.43 to 1.56; *p* = 0.46; *I* ^2^ = 32%; very low certainty; Figure 7) and (MD = −0.71; 95% CI = −2.30 to 0.88; *p* = 0.38; *I* ^2^ = 0%; low certainty; Figure 8), respectively.

**Figure 9:**
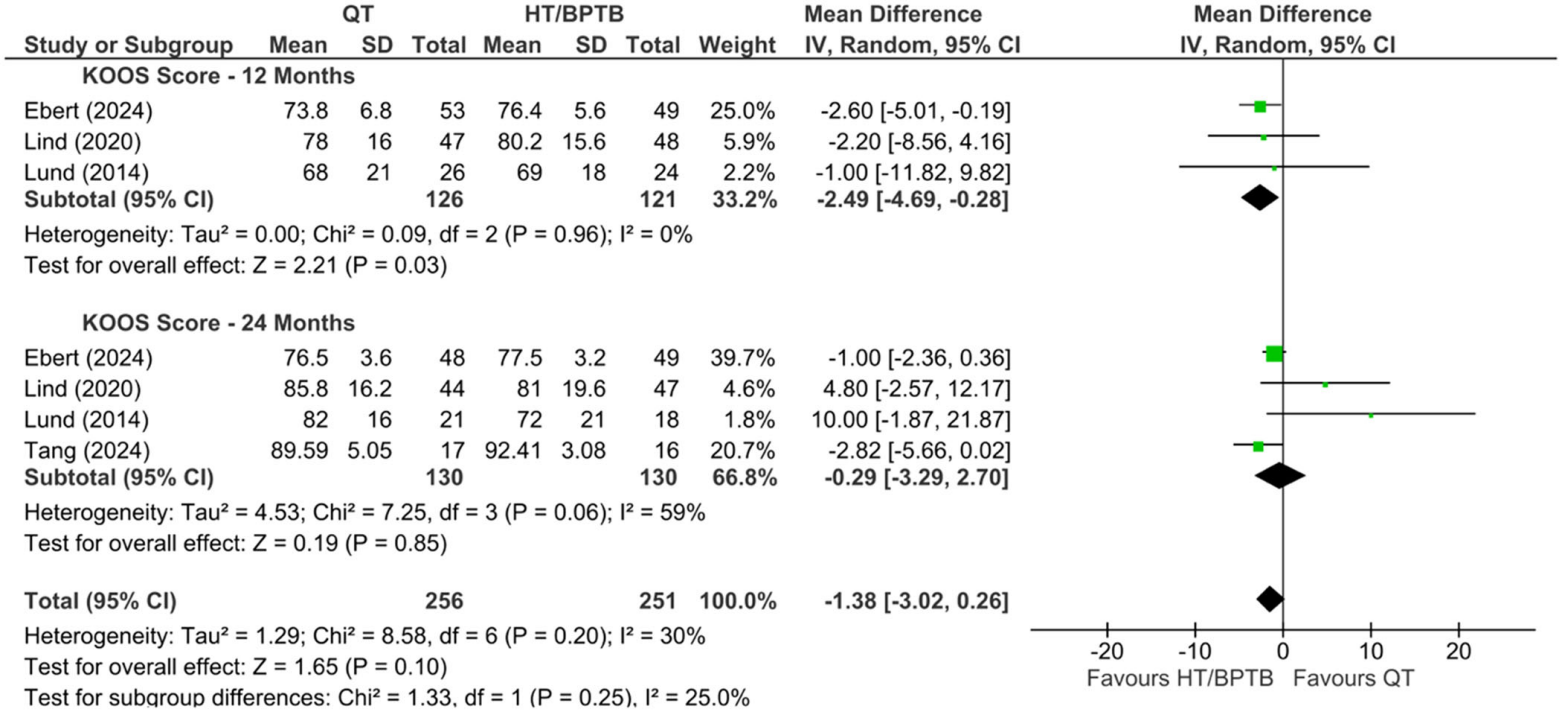
Meta‐analysis comparing KOOS scores in hamstring tendon (HT) and bone‐patellar tendon‐bone (BPTB) versus quadriceps tendon (QT) at 12 and 24 months. KOOS, Knee injury and Osteoarthritis Outcome Score.

A comparison of QT versus HT and/or BPTB autograft for the Knee injury and Osteoarthritis Outcome Score (KOOS) was reported in four studies [6, 23, 56], at 12 and 24 months. At 12 months, three studies [23, 56, 60, 94] demonstrated an overall significant difference, favouring HT and BPTB over QT autografts (MD = −2.49; 95% CI = −4.69 to −0.28; *p* = 0.03; *I* ^2^ = 0%; very low certainty; Figure 9). At 24 months, there was no significant difference when comparing QT to HT and BPTB autografts (MD = −0.29; 95% CI = −3.29 to 2.70; *p* = 0.85; *I* ^2^ = 59%; very low certainty; Figure 9). Collectively, at 12 and 24 months, the pooled effect illustrated similar outcomes for QT when compared to HT and BPTB autografts (MD = −1.38; 95% CI = −3.02 to 0.26; *p* = 0.10; *I* ^2^ = 30%; very low certainty; Figure 9).

**Figure 10:**
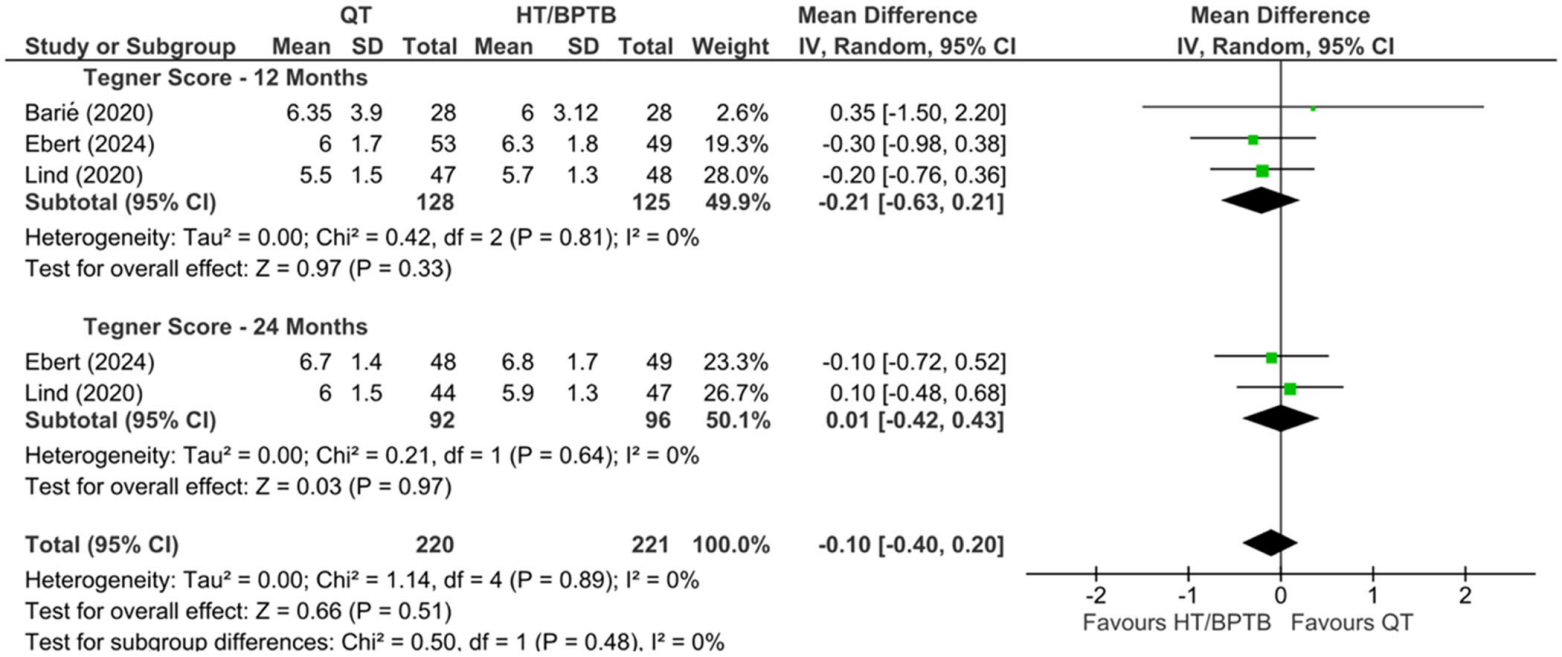
Meta‐analysis comparing Tegner scores in hamstring tendon (HT) and bone‐patellar tendon‐bone (BPTB) versus quadriceps tendon (QT) at 12 and 24 months.

A comparison of QT versus HT and/or BPTB autograft for the Tegner Score was reported in three studies [23, 56, 60], at 12‐ and 24‐month periods. The overall effect showed that QT demonstrated similar return to sport outcomes when compared to HT and BPTB with no significant differences at both 12 and 24 months (MD = −0.10; 95% CI = −0.40 to 0.20; *p* = 0.51; *I* ^2^ = 0%; high certainty; Figure 10).

## DISCUSSION

This systematic review with meta‐analysis demonstrates that QT autografts provide comparable clinical outcomes to traditional HT and BPTB autografts for ACL reconstruction, with some evidence to suggest lower donor site morbidity. Meta‐analysis of six RCTs [6, 26, 42, 60, 64, 69] revealed no significant differences in failure rates, consistent with previous reviews [1, 7, 53, 70, 75, 86, 95]. However, the interpretation of graft outcomes is complicated by inconsistent terminology across studies. While ‘graft failure’, ‘retear’ and ‘re‐injury’ are often used interchangeably, they may represent different clinical scenarios. For example, four studies [6, 60, 64, 69] specifically documented ACL retears resulting from traumatic sporting incidents, whereas two studies [26, 42] did not specify the mechanism of graft failure. This lack of standardised definitions and reporting of injury mechanisms makes it difficult to accurately compare re‐rupture rates between studies and may explain the varying rates reported in the literature.

Graft failure is often associated with key biomechanical properties, including graft size, tensile strength, and stiffness [5, 69, 77, 85, 86]. These properties can vary due to surgical factors such as graft preparation techniques, tunnel position and fixation methods, along with patient factors such as age, sex, body mass index (BMI) and lifestyle factors [5, 27]. Typical graft CSA for QT, HT and BPTB grafts are 91.2, 57 and 48.4 mm^2^, respectively [5]. In many previous studies, lower graft diameter has been shown to be a predictor for graft revision [61, 66, 75, 79]. Conversely, larger grafts carry a higher risk of complications such as arthrofibrosis [92] and graft impingement [95].

The average tensile strength, otherwise reported as load until failure, is 2186, 2422 and 1581–1784 N for QT, HT and BPTB autografts, respectively [5, 86]. While the tensile strength of the native ACL is approximately 2160 N [5, 62]. This closely aligns with the QT value, suggesting adequate mechanical strength for ACLR. QT grafts also contain up to 20% more collagen fibres and a higher density of fibroblasts compared to BPTB, potentially supporting better graft integration [34, 58, 77]. Additionally, QT grafts can be harvested with a single bone block, which may enhance early graft healing [9, 58, 77]. These differences in graft size and tensile strength are an important consideration for graft selection, as higher tensile strength and diameter may offer greater resistance to failure under high‐load activities.

Stiffness, referring to the graft's ability to resist elongation when force is applied [67], also varies among graft types. Quadriceps tendon, HT, and BPTB autografts have an average stiffness of 466, 238 and 210–278 N/mm, respectively [5, 54, 86]. Graft elongation may contribute to post‐operative anteroposterior (AP) laxity, which increases the risk of re‐rupture [71, 102]. Despite these biomechanical differences, this review did not identify significant differences in post‐operative AP knee laxity between graft types, as measured by the KT‐1000 arthrometer. This is in line with previous systematic reviews [1, 17, 69, 89] in which they found no differences in laxity between graft types. Persisting pivot shift following ACLR implies ongoing rotational laxity and is a concern for re‐injury risk upon return to sport. This may be due to graft characteristics but may also be due to tunnel position and graft fixation techniques. We found no difference in rotational laxity with similar pivot shift testing between the different grafts.

Regarding donor site morbidity, a difference was identified between QT and HT/BPTB autografts, with QT autografts resulting in reduced post‐operative harvest site pain, kneeling pain, and squatting pain compared to BPTB and HT autografts. This aligns with literature demonstrating the increased incidence of anterior knee pain with BPTB autografts compared to QT [17, 50, 69, 89]. Other reviews have found that QT autografts significantly reduce donor site morbidity compared to HT autografts [17, 43], while some reviews reported no differences between QT and HT autografts [50, 69]. Inconsistencies in the findings may stem from differing graft harvest techniques and rehabilitation protocols as well as the varying measures of symptoms used in these studies.

When harvesting at the anteromedial aspect of the knee for HT and BPTB autografts, there is a greater risk for cutaneous nerve injury compared to QT autografts [3, 15, 17, 35, 47, 49]. In ACLR, these iatrogenic nerve injuries can result in numbness, paraesthesia and/or neuropathic pain, thus contributing to increased donor site morbidity [4, 49, 96]. Studies have shown that HT autografts commonly involve risk to the saphenous nerve and its infra‐patella branches, while BPTB autografts are particularly associated with injury to the infra‐patella branch of the saphenous nerve [15, 29, 36, 41]. Alternatively, the QT autograft may only result in the injury of the less significant intermediate cutaneous branches from the femoral nerve [17, 41, 62]. The overall incidence of nerve injury with QT autografts is lower when compared to HT and BPTB autografts due to the nature of the harvest location, making them a favourable option for minimising nerve‐related donor site morbidity [3, 17, 33, 50, 69, 83]. The clinical implications of donor site morbidity are significant. Reduced donor site morbidity can lead to faster post‐operative recovery, improved patient satisfaction and potentially better long‐term function, particularly in patients who need to kneel or squat frequently for work or daily activities [57, 69, 78].

The harvest site additionally has a direct impact on the knee's surrounding musculature and subsequent extension or flexion deficits [15, 90]. This review identified that QT autografts are associated with notable extensor weakness, which may persist for 12–24 months, whereas HT autografts predominantly lead to flexion weakness. This finding aligns with observations previously reported [37, 80, 82]. These muscle‐specific weaknesses can contribute to downstream functional impairments. For QT and autografts, the prolonged extensor weakness observed may impair the restoration of the post‐operative knee extension range of motion [42, 45]. This pattern is also observed with BPTB grafts, which also show greater quadriceps weakness compared to HT grafts [98]. Additionally, female sex, higher BMI and loss of extension range of motion are independent predictors of reduced extensor strength [42, 98]. These are important considerations, as limited knee extension is a well‐documented risk factor for the development of arthrofibrosis, a complication that can restrict knee function and delay rehabilitation progress [22, 28, 73].

In ACLR utilising HT autografts, the observed reduction in flexion strength is clinically relevant, particularly due to its impact on the HQ ratio. Within this review, studies by Martin‐Alguacil et al. [64] and Tang et al. [94] reported that HT autografts were associated with a significantly lower HQ ratio compared to QT autografts. This is a comparable finding across other studies [9, 24, 80]. A diminished HQ ratio has been linked to increased strain on the ACL and a heightened risk of reinjury [30, 48, 70, 76]. This emphasises the importance of implementing graft‐specific rehabilitation protocols tailored to address the unique biomechanical deficits associated with each graft type in ACLR.

This review demonstrates that patients who have undergone a primary ACLR will achieve comparable subjective outcomes, regardless of autograft type used, by 12 and 24 months post‐operatively, which supports existing findings [1, 17, 43, 80, 89]. Similar results in PROMs suggest that despite differences in biomechanical properties, donor site morbidity, and specific rehabilitation challenges associated with each autograft, patients ultimately achieve comparable subjective outcomes [2, 65, 80]. This underscores the efficacy of all three autograft options in restoring function and quality of life after ACLR. Importantly, these findings empower shared decision‐making between clinicians and patients, as the choice of graft can be personalised to individual needs, anatomical considerations and activity levels without compromising overall functional outcomes.

### Strengths and limitations

The strengths of this review include methodological rigour, adherence to PRISMA guidelines and the inclusion criteria for RCT study design, ensuring a high level of evidence [87]. However, limitations include the relatively small sample size (636 participants), reflecting the exclusion of cohort studies. Randomised graft allocation in RCTs may not account for individual patient needs and anatomical differences, limiting applicability to clinical practice. Furthermore, shorter‐term follow‐ups restrict the assessment of long‐term outcomes, such as changes in graft failure rates, graft laxity, patient strength and the development of osteoarthritis [16, 19]. Using the GRADE approach, this review found high‐certainty evidence for graft failure, donor site morbidity and Tegner scores, as well as moderate certainty for KT‐1000 and Pivot Shift measurements. Certainty for other outcomes was low due to heterogeneity, wide CIs, and moderate sample sizes. Finally, comparing QT grafts against combined HT and BPTB grafts in meta‐analyses, rather than separately, means that direct conclusions for QT versus HT or QT versus BPTB cannot be made in isolation.

### Future considerations

RCTs with larger population sizes and well‐designed observational cohort studies with extended follow‐up periods are necessary to determine whether there are significant differences in graft failure rates between QT, HT and BPTB autografts. Future research should prioritise the standardised reporting of graft failure and complications, enabling better data pooling for meta‐analyses and more accurate comparisons across studies. Arthrofibrosis rates in the three autograft choices require further investigation, especially considering the QT autograft's larger CSA and its potential impact on knee extension strength and range of motion deficits. Additionally, standardised measures for donor site morbidity should be developed and universally adopted to improve the comparability of studies, allowing for repeatable evaluation. Collectively, addressing these research gaps will facilitate more evidence‐based decision‐making in graft selection and hopefully improve long‐term outcomes for patients undergoing ACLR.

## CONCLUSION

Quadriceps tendon autografts for ACL reconstruction have similar outcomes to HT and BPTB autografts when assessing graft failure, complications, and laxity. Quadriceps tendon autografts cause lower donor site morbidity when compared to BPTB and HT autografts. While each graft type is associated with specific complications and post‐operative strength deficits, all three remain viable options for ACLR with similar patient‐reported outcomes and return to sport. This study demonstrates that each of the grafts can result in good outcomes and supports individualised graft selection, with consideration of the patient's functional demands, anatomy and rehabilitation goals.

## AUTHOR CONTRIBUTIONS

**Ty White**: Methodology; investigation; data curation; formal analysis; writing—original draft; writing—review and editing, visualisation. **Matthew Castro**: Investigation; data curation; validation; writing—original draft; writing—review and editing; visualisation. **Lucas Antonio**: Investigation; data curation; validation; writing—original draft; writing—review and editing; visualisation. **Wayne Hing**: Supervision; resources; methodology; writing—review and editing; project administration. **Francois Tudor**: Conceptualisation; supervision; methodology; writing—review and editing. **Larissa Sattler**: Conceptualisation; supervision; resources; methodology; writing—review and editing; project administration.

## CONFLICT OF INTEREST STATEMENT

The authors declare no conflicts of interest.

## ETHICS STATEMENT

Ethics approval was not required for this systematic review as it synthesised existing published literature and did not involve the collection of primary data from human participants.

## Supporting information

Supplementary Material.

## Data Availability

All data analysed in this systematic review are from published studies cited within this manuscript.
