# Supplementary material for: Quadriceps, hamstring and patella tendon autografts for primary anterior cruciate ligament reconstruction demonstrate similar clinical outcomes, including graft failure, joint laxity and complications: A systematic review with meta‐analysis of randomised controlled trials
Source: Knee Surg Sports Traumatol Arthrosc. 2025 Jul 18;34(5):1631–46. doi: 10.1002/ksa.12755 (PMC13122740; doi:10.1002/ksa.12755)
Supplement: Supplementary file 1 — Supplementary Material. [file KSA-34-1631-s001.docx]

**Supplementary Material**

*Appendix 1. Database search strategies*

| **Database** | **Search strategy** |
| --- | --- |
| **PubMed** | ("Anterior Cruciate Ligament"[MeSH Terms] OR "Anterior Cruciate Ligament"[Title/Abstract] OR "ACL"[Title/Abstract]) AND ("hamstring*"[Title/Abstract] OR "semitendinos*"[Title/Abstract] OR "gracilis"[Title/Abstract] OR "Patella Tendon"[Title/Abstract] OR "BPTB"[Title/Abstract]) AND "quad*"[Title/Abstract] |
| **Cochrane** | ([mh "Anterior Cruciate Ligament"] OR "Anterior Cruciate Ligament":ti,ab OR ACL:ti,ab) AND (hamstring*:ti,ab OR semitendinos*:ti,ab OR gracilis:ti,ab OR "Patella Tendon":ti,ab OR BPTB:ti,ab) AND quad*:ti,ab |
| **EMBASE** | ('Anterior Cruciate Ligament'/exp OR 'Anterior Cruciate Ligament':ti,ab OR ACL:ti,ab) AND (hamstring*:ti,ab OR semitendinos*:ti,ab OR gracilis:ti,ab OR 'Patella Tendon':ti,ab OR BPTB:ti,ab) AND quad*:ti,ab |
| **SPORT-Discus** | (DE "Anterior Cruciate Ligament" OR (TI "Anterior Cruciate Ligament" OR AB "Anterior Cruciate Ligament") OR (TI "ACL" OR AB "ACL")) AND ((TI "hamstring*" OR AB "hamstring*") OR (TI "semitendinos*" OR AB "semitendinos*") OR (TI "gracilis" OR AB "gracilis") OR (TI "Patella Tendon" OR AB "Patella Tendon") OR (TI "BPTB" OR AB "BPTB")) AND (TI "quad*" OR AB "quad*") |
| **CINHAL** | ((MH "Anterior Cruciate Ligament+") OR (TI "Anterior Cruciate Ligament" OR AB "Anterior Cruciate Ligament") OR (TI ACL OR AB ACL)) AND ((TI hamstring* OR AB hamstring*) OR (TI semitendinos* OR AB semitendinos*) OR (TI gracilis OR AB gracilis) OR (TI "Patella Tendon" OR AB "Patella Tendon") OR (TI BPTB OR AB BPTB)) AND (TI quad* OR AB quad*) |

*Appendix 2:* *Patient Demographics*

| **Author, Year & Country** | **Graft Type** | | | **Assessment Timepoints (Months)** |
| --- | --- | --- | --- | --- |
|  | Quadriceps Tendon (QT)    No. of participants  Sex (M/F)  Mean age (SD) | Hamstring Tendon (HT)    No. of participants  Sex (M/F)  Mean age (SD) | Bone-Patella-Tendon-Bone (BPTB)  No. of participants  Sex (M/F)  Mean age (SD) |  |
| **Barié, 2020, Germany** | 30  17/13  30.5 (7.8) |  | 30  17/13  30.6 (7.5) | 0, 12, 120 |
| **Buescu, 2017, Romania** | 24  19/5  29.21 (8.52) | 24  19/5  27.5 (5.62) |  | 0-12^†^, 23-24^†^, 25-48^†^ |
| **Ebert, 2024, Australia** | 57  28/29  28.1 (8.2) | 55  28/27  29.4 (7.7) |  | 0, 1.5, 3, 6, 12, 24 |
| **Horstmann, 2020, Germany** | 24  21/3  24.1 (3.6) | 27  12/15  32.7 (11.4) |  | 0, 3, 6, 12, 24 |
| **Lind, 2020, Denmark**^#^ | 50  29/21  27.2 (6.4) | 49  25/24  27.1 (6.1) |  | 12, 24 |
| **Lund, 2014, Denmark** | 26  21/5  30 (9) |  | 25  21/4  31 (8) | 12, 24 |
| **Martin-Alguacil, 2019, Spain^*^** | 26  23/3  18.7 (3.6) | 25  16/9  19.2 (3.6) |  | 0, 3, 6, 12 |
| **Martin-Alguacil, 2018, Spain^*^** | 26  23/3  18.7 (3.6) | 25  16/9  19.2 (3.6) |  | 0, 3, 6, 12, 24 |
| **Sinding, 2020, Denmark**^#^ | 42  25/17  28.7 (6.4) | 43  23/20  28.3 (6.2) |  | 12 |
| **Tang, 2024, Turkey** | 20  NR  28.06 (6.24) | 20  NR 28.31 (8.55) |  | 0, 24 |
| **Tirupathi, 2019, India** | 48  NR  NR | 48  NR  NR |  | 0, 6, 12, 24 |
| **Vilchez-Cavazos, 2020, Mexico** | 14  11/3  23^‡^ | 14  12/2  23^‡^ |  | 0, 12 |

^†^Indicates hour(s) as assessment timepoint

^#^Uses the same study population

^*^Uses the same study population

NR, data not reported

^‡^Data shown as median

*Appendix 3: Study Inclusion and Exclusion Criteria*

| **Author, Year & Country** | **Inclusion Criteria** | **Exclusion Criteria** |
| --- | --- | --- |
| **Barié, 2020, Germany** | 1. Primary ACL rupture 2. Activity level with a minimum score of 6 on the Tegner scale | 1. ACL re-rupture 2. Concomitant instability of the PCL or lateral instability 3. The need for subtotal meniscal resection 4. Presence of knee osteoarthritis stage 3 or 4 according to Kellgren-Lawrence score 5. Cartilage lesions greater than grade 2 in the Outerbridge classification |
| **Buescu, 2017, Romania** | 1. Patients between 16 to 50 years of age with a documented ACL tear scheduled for arthroscopic reconstruction 2. Willing to participate in the study | 1. Associated meniscus tears requiring suture repair 2. Associated ligamentous lesions requiring surgical management 3. Previous surgeries on the same knee 4. Patients with chronic pain 5. Hepatic impairment 6. Alcohol / drug abuse 7. Analgesic use within 14 days of admission 8. Patients who refused to participate in the study |
| **Ebert, 2024, Australia** | 1. Patients between 16 to 50 years of age 2. Qualification for ACLR based on clinical examination and magnetic resonance imaging | 1. Unable or unwilling to sign informed consent specific to the study 2. Body mass index ≥ 40 3. Revision or multi-ligamentous reconstruction 4. Presentation of significant articular cartilage pathology requiring concomitant surgical intervention 5. Receiving treatment for a psychiatric disorder (senile dementia, Alzheimer disease) 6. Alcohol / substance abuse |
| **Horstmann, 2020, Germany** | 1. Eligible subjects with ACL insufficiency as determined by subjective instability and giving way episodes, clinical examination, and MRI scan 2. Subjects between 18-50 years of age | 1. ACL insufficiency after reconstruction (revision surgery) 2. Posterior cruciate ligament insufficiency 3. Complete posterolateral corner injury 4. Loss of more than two thirds of at least one meniscus during surgery 5. Full-thickness cartilage lesion 6. Fractures |
| **Lind, 2020, Denmark** | 1. MRI documented ACL lesion with subjective instability symptoms | 1. Active malignant disease 2. Other knee ligament instability 3. Rheumatoid arthritis 4. Morbus Bechterew 5. Body mass index > 30 6. Current treatment with glucocorticoid drugs or growth hormone 7. Expected inability to complete the standard rehabilitation program |
| **Lund, 2014, Denmark** | 1. Isolated ACL injuries in adults > 18 years of age in which the surgeon chose a BPTB graft for reconstruction | 1. Previous ligament reconstruction 2. Multiple ligament injuries defined as ACL injuries 3. Requirement for further repair to the posterior cruciate ligament or collateral ligaments 4. Objective laxity of IKDC grade 2 or higher following brace treatment 5. Bilateral ACL injuries 6. Concomitant articular cartilage lesions larger than 2 cm^2^ with a depth of more than 50% of the cartilage thickness 7. Meniscal injuries involving more than two-thirds of the meniscus or the need for meniscus repair |
| **Martin-Alguacil, 2019, Spain** | 1. Confirmed ACL rupture 2. Surgery carried out less than 6 months after the injury 3. Being recreational or federated athletes | 1. Having a previous knee injury or surgery 2. Concomitant ligament injuries and/or meniscal tear 3. Joint cartilage lesions greater than Outerbridge grades III-IV |
| **Martin-Alguacil, 2018, Spain** | 1. Having suffered an ACL injury with less than six months of evolution of the lesion at the time of diagnosis 2. Competitive soccer players | 1. Previous joint injury or surgery on the affected knee 2. If they had any concomitant ligament injury 3. Removal of more than 50% of either lateral or medial meniscus 4. Articular cartilage lesion greater than Outerbridge grades I-II |
| **Sinding, 2020, Denmark** | 1. Patients over the age of 18 years scheduled for isolated ACL-R | 1. Diabetes, arthritis, active malignant disease, other ligamentous instability, Morbus Bechterew, other disabling musculoskeletal illness 2. BMI > 30 3. Current treatment with glucocorticoid drugs or growth hormone 4. Expected inability to complete the standard rehabilitation program |
| **Tang, 2024, Turkey** | 1. Having an isolated primary ACL injury 2. Willing to join the study 3. Between 18 and 50 years of age 4. No previous surgery in the injured knee | 1. Multi-ligamentous injuries 2. Revision cases 3. Chondral lesions larger than Outerbridge grade 2 4. Individuals younger than 18 and older than 50 |
| **Tirupathi, 2019, India** | 1. Unilateral isolated ACL injuries | 1. Previous surgeries in the involved knee 2. Significant deformities of the involved knee 3. Associated major comorbidities limiting postoperative rehabilitation protocol |
| **Vilchez-Cavazos, 2020, Mexico** | 1. Patients > 18 years of age 2. Agreement to participate in study 3. Clinical and medical resonance imaging diagnosis of an ACL rupture 4. With or without meniscal lesion 5. No previous knee surgery | 1. Multi-ligament lesions 2. Osteoarthritis 3. Chronic degenerative diseases 4. Rheumatoid arthritis 5. ACL contralateral lesions |

*Appendix 4: Primary Outcome (Graft Failure)*

| **Author, Year** | **Graft Type** | | |
| --- | --- | --- | --- |
|  | Quadriceps Tendon (QT)  Graft Failures / Total Participants (n=) | Hamstring Tendon (HT)  Graft Failures / Total Participants (n=) | Bone-patella-tendon-bone (BPTB)  Graft Failures / Total Participants (n=) |
| **Barié *et al.*, 2020** ^[6]^ | 0/30 |  | 1/30 |
| **Ebert *et al.*, 2024** ^[23]^ | 1/57 | 0/55 |  |
| **Horstmann *et al.,* 2020^[40]^** | 3/24 | 1/27 |  |
| **Lind *et al.,* 2020**^[56]^ | 1/50 | 1/49 |  |
| **Lund *et al.,* 2014**^[60]^ | 0/26 |  | 1/25 |
| **Martin-Alguacil *et al.,* 2018** ^[64]^ | 1/28 | 3/28 |  |
| **Total**  **%** | 6/215  2.79% | 5/159  3.14% | 2/55  3.64% |

Studies that report on graft failure are included within the table.

*Appendix 5: Secondary Outcome Measures*

| **Author, Year** | **Outcome Measure (s)** | **Measurement Tool (s)** | **Timepoint of Assessments (months)** | **P value (<0.05)   Timepoint (months)** |
| --- | --- | --- | --- | --- |
| **Barié *et al.*, 2020** | 1. Patient Reported Outcomes 2. Clinical Outcomes | 1a. Lysholm Score  1b. Tegner Score  1c. IKDC Score  1d. Donor Site Morbidity (IKDC Sub-score 9c/9d)  1e. Satisfaction  2a. KT-1000 | 0 months  12 months  120 months | 12 months  1d. *p* < 0.001^*^ / *p* = 0.003^*^  120 months  1d. *p* = 0.019^*^ / *p* = 0.046^*^ |
| **Buescu *et al.*, 2017** | 1. Patient Reported Outcomes 2. Clinical Outcomes | 1a. Pain VAS  2a. Time to first analgesic requirement  2b. Number of doses Tramadol | 0-12hrs  13-24hrs  25-48hrs | 0-48hrs  2b. *p* = 0.009^*^, *p* = 0.002^*^ |
| **Ebert *et al.*, 2024** | 1. Patient Reported Outcomes 2. Clinical Outcomes 3. Functional Outcomes | 1a. Lysholm Score  1b. Tegner Score  1c. IKDC Score  1d. KOOS  1e. Cincinnati  1f. VAS-knee  1g. VAS – Graft  1h. ACL-RSI  1i. Donor Site Score  1j. GRC  2a. KT-1000  2b. Range of Motion  2c. LSI - Peak Knee Extensor Torque  2d. LSI - Peak Knee Flexor Torque  3a. LSI - Single hop for Distance  3b. LSI - 6-meter timed hop  3c. LSI- Triple hop for distance  3d. LSI – Triple cross over hop for distance  3e. LSI – Single medial hop for distance  3f. LSI – Single lateral hop for distance | 0 months  1.5 months  3 months  6 months  12 months  24 months | 0-24 months  1h. *p* = 0.040^^^  6, 12 months  2c. *p* = 0.003^^^  6,12, 24 months  2d. *p* < 0.001^*^  3a. *p* = 0.026^^^  3e. *p* = 0.007^^^  3f. *p* = 0.046^^^ |
| **Horstmann *et al.,* 2020** | 1. Patient Reported Outcomes 2. Clinical Outcomes 3. Functional Outcomes | 1a. Lysholm Score  1b. IKDC Score  2a. KT-1000  2b. Pivot Shift  3a. Return to Work  3b. Return to Sport  3c. Extension Max Strength  3d. Flexion Max Strength | 0 months  3 months  6 months  12 months  24 months | 0 months  1a. *p* = 0.010^*^ |
| **Lind *et al.,* 2020** | 1. Patient Reported Outcomes 2. Clinical Outcomes 3. Functional Outcomes | 1a. IKDC Score  1b. KOOS  1c. Tegner Score  1d. Kujala Score  1e. Donor Site Morbidity Score  2a. KT-1000  2b. Pivot Shift Test  3a. One-leg hop test | 12 months  24 months | 12 months  3a. *p* = 0.02^^^  24 months  1e. *p* < 0.04^*^ |
| **Lund *et al.,* 2014** | 1. Patient Reported Outcomes 2. Clinical Outcomes | 1a. IKDC Score  1b. KOOS  1c. Kneeling pain  1d. Harvest site pain  1e. Tibial Sensory loss  2a. KT-1000  2b. Pivot Shift | 12 months  24 months | 12 months  1c. *p* = 0.03^*^  1d. *p* = 0.02^*^  2b. *p* = 0.03^*^ |
| **Martin-Alguacil *et al.,* 2019** | 1. Patient Reported Outcomes 2. Clinical Outcomes | 1a. Pressure Pain Threshold  2a. Muscle architecture via Ultrasound | 0 months  3 months  6 months  12 months | ND |
| **Martin-Alguacil *et al.,* 2018** | 1. Patient Reported Outcomes 2. Clinical Outcomes | 1a. Tegner Score  1b. Cincinnati Score  2a. KT-2000  2b. H/Q Ratio  2c. Flexion Strength  2d. Extension Strength | 0 months  3 months  6 months  12 months  24 months | 3 months  2b. *p* = 0.003^*^  2d. *p* = 0.014^^^  6 months  2b. *p* = 0.0012^*^  2d. *p* = 0.004^^^  12 months  2b. *p* = 0.005^*^  2d. *p* = 0.04^^^ |
| **Sinding *et al.,* 2020** | 1. Patient Reported Outcomes 2. Clinical Outcomes 3. Functional Outcomes | 1a. IKDC Score  2a. LSI – Knee Extension  2b. LSI – Knee Flexion  2c. H/Q Ratio  3a. LSI – Single leg hop test | 12 months | ND |
| **Tang *et al.,* 2024** | 1. Patient Reported Outcomes 2. Clinical Outcomes | 1a. Lysholm Score  1b. IKDC Score  1c. KOOS  2a. Lachman  2b. Anterior Drawer  2c. Extensor Peak Torque (%BW/Nm)  2d. Flexor Peak Torque (%BW/Nm)  2e. Thigh Circumference  2f. H/Q Ratio | 0 months  24 months | 24 months  2c. *p* = 0.001^^^ / *p* = 0.003^^^  2f. *p* < 0.05^*^ |
| **Tirupathi *et al.,* 2019** | 1. Patient Reported Outcomes | 1a. IKDC Score | 0 months  6 months  12 months  24 months | ND |
| **Vilchez-Cavazos *et al.,* 2020** | 1. Patient Reported Outcomes 2. Clinical Outcomes | 1a. Lysholm Score  1b. IKDC Score  1c. VAS – Pain  1d. SF-12  2a. Range of Motion – Flexion  2b. Range of Motion - Extension | 0 months  12 months | ND |

^*^ Favours QT Autograft

^^^ Favours HT/BPTB Autograft

ND, No Difference

All Outcomes that are not presented within the last column of the table showed no significant difference between groups. Only significant differences were listed.

*Appendix 6: Complications and Reoperations*

| **Author, Year** | | | | | | | | | | | | | | | | | | | | | | | | | | |
| --- | --- | --- | --- | --- | --- | --- | --- | --- | --- | --- | --- | --- | --- | --- | --- | --- | --- | --- | --- | --- | --- | --- | --- | --- | --- | --- |
|  | **Barié et al., 2020** | | | | **Lind et al., 2020** | | | **Lund et al., 2014** | | | **Ebert et al., 2024** | | | **Horstmann et al., 2020** | | | **Tang et al., 2024** | | | | **Total** | | | | | |
|  | **QT** | | **HT** | **BPTB** | **QT** | **HT** | **BPTB** | **QT** | **HT** | **BPTB** | **QT** | **HT** | **BPTB** | **QT** | **HT** | **BPTB** | **QT** | **HT** | **BPTB** | | **QT** | | **HT** | | | **BPTB** |
| ***Complications*** |  | |  | | | | | | | | | | | | | | | | | | | ***Events / Total group participants (%)*** | | | | |
| Cyclopes  Lesion | 1/30 |  | | 3/30 | 4/50 | 3/49 |  |  |  |  |  |  |  |  |  |  |  |  |  | | 5/80 | | 3/49 | | | 3/30 |
| Post-operative stiffness (MUA) |  |  | |  |  |  |  |  |  |  |  | 1/55 |  |  |  |  |  |  |  | |  | | 1/55 | | |  |
| Infection |  |  | |  |  |  |  |  |  |  |  |  |  | 1/24 |  |  |  |  |  | | 1/24 | |  | | |  |
| IPBSN Palsy |  |  | |  |  |  |  |  |  |  |  |  |  |  |  |  |  | 4/20 |  | |  | | 4/20 | | |  |
| Hematoma |  |  | |  |  |  |  |  |  |  |  |  |  |  |  |  | 2/20 |  |  | | 2/20 | |  | | |  |
| ***Reoperations*** |  |  | |  |  |  |  |  |  |  |  |  |  |  |  |  |  |  |  | |  | |  | | |  |
| Scar tissue debridement |  |  | |  |  |  |  |  |  |  | 1/57 | 2/55 |  |  |  |  |  |  |  | | 1/57 | | 2/55 | | |  |
| Meniscal Repair | 2/30 |  | | 3/30 |  | 1/49 |  |  |  |  |  | 1/55 |  |  |  |  |  |  |  | | 2/30 | | 2/104 | | | 3/30 |
| Osteochondral Autologous Transplant |  |  | |  |  |  |  |  |  |  | 1/57 |  |  |  |  |  |  |  |  | | 1/57 | |  | | |  |
| Partial Meniscectomy |  |  | |  |  |  |  |  |  | 1/25 |  |  |  |  |  |  |  |  |  | |  | |  | | | 1/25 |
| Plica Resection |  |  | |  |  |  |  | 1/26 |  |  |  |  |  |  |  |  |  |  |  | | 1/26 | |  | | |  |
| Tibial Screw Removal |  |  | |  |  | 1/49 |  | 1/26 |  | 1/25 |  |  |  |  |  |  |  |  |  | | 1/26 | | 1/49 | | | 1/25 |
| Removal of staple |  |  | |  |  |  |  |  |  |  |  |  |  |  |  |  |  | 1/20 |  | |  | | 1/20 | | |  |
| Notch debridement |  |  | |  |  |  |  |  |  |  | 1/57 | 1/55 |  |  |  |  |  |  |  | | 1/57 | | 1/55 | | |  |
| ***Total*** |  | |  | | | | | | | | | | | | | | | | | ***15/377*** | | | | ***15/407*** | ***8/110*** | |

IPBSN – Infrapatellar branch of the saphenous nerve
